# Circular RNA SCMH1 suppresses KMO expression to inhibit mitophagy and promote functional recovery following stroke

**DOI:** 10.7150/thno.99323

**Published:** 2024-10-28

**Authors:** Yu Wang, Ying Bai, Yang Cai, Yuan Zhang, Ling Shen, Wen Xi, Zhongqiu Zhou, Lian Xu, Xue Liu, Bing Han, Honghong Yao

**Affiliations:** 1 Jiangsu Provincial Key Laboratory of Critical Care Medicine, Department of Pharmacology, School of Medicine, Southeast University, Nanjing, China.; 2 Co-innovation Center of Neuroregeneration, Nantong University, Nantong, China.; 3 Institute of Life Sciences, Key Laboratory of Developmental Genes and Human Disease, Southeast University, Nanjing, China.

**Keywords:** circSCMH1, functional recovery, ischemic stroke, KMO, STAT5B

## Abstract

**Rationale:** Metabolic dysfunction is one of the key pathological events after ischemic stroke. Disruption of cerebral blood flow impairs oxygen and energy substrate delivery, leading to mitochondrial oxidative phosphorylation dysfunction and cellular bioenergetic stress. Investigating the effects of circSCMH1, a brain repair-related circular RNA, on metabolism may identify novel therapeutic targets for stroke treatment.

**Methods:** CircSCMH1 was encapsulated into brain-targeting extracellular vesicles (EVs) mediated by rabies virus glycoprotein (RVG). Using a mouse model of photothrombotic (PT) stroke, we employed metabolomics and transcriptomics, combined with western blotting and behavioral experiments, to identify the metabolic targets regulated in RVG-circSCMH1-EV-treated mice. Additionally, immunofluorescence staining, chromatin immunoprecipitation (ChIP), pull-down, and western blotting were utilized to elucidate the underlying mechanisms.

**Results:** The targeted delivery of circSCMH1 via RVG-EVs was found to promote post-stroke brain repair by enhancing mitochondrial fusion and inhibiting mitophagy through suppression of kynurenine 3-monooxygenase (KMO) expression. Mechanistically, circSCMH1 exerted its inhibitory effect on KMO expression by binding to the transcription activator STAT5B, thereby impeding its nuclear translocation.

**Conclusions:** Our study reveals a novel mechanism by which circSCMH1 downregulates KMO expression, thereby enhancing mitochondrial fusion and inhibiting mitophagy, ultimately facilitating post-stroke brain repair. These findings shed new light on the role of circSCMH1 in promoting stroke recovery and underscore its potential as a therapeutic target for the treatment of ischemic stroke.

## Introduction

Ischemic stroke is a leading cause of adult disability, characterized by neurological deficits caused by cerebrovascular occlusion 1-3. At present, therapeutic agents to promote stroke recovery and improve long-term outcomes are lacking 4. High levels of oxygen and glucose are essential for the brain, due to its high intrinsic metabolic activity 5. Growing evidence demonstrates that metabolic reprogramming after stroke improves long-term neurological function and is strongly associated with high survival rates in animal models and stroke patients 6, 7. Efforts to explore therapeutic approaches to promoting post-stroke metabolic reprogramming are vital areas of research that have provided benefits to many ischemic stroke patients.

Among various molecules, circular RNAs (circRNAs) have garnered increasing attention in recent years for their role in regulating various metabolic pathways, such as glucose, lipid, and amino acid metabolism 8-10. As one type of endogenous non-coding RNA molecules, circRNAs are characterized by their covalently closed continuous loops formed through back-splicing 11-13. Previous studies have shown that circRNAs are widely distributed in the brain, and several of them, such as circHECTD1, circDLGAP4, circTLK1, circFoxO3, and circSCMH1, have been functionally linked to ischemic stroke 14-18. This revealed that developing therapeutic drugs targeting circRNAs was a viable direction for the treatment of ischemic stroke.

In our previous study, we discovered that circSCMH1 was expressed in various cell types in the brain and promoted post-stroke brain repair by enhancing neuronal plasticity and facilitating vascular repair 18, 19. To increase the clinical translational value of circSCMH1 research, we utilized extracellular vesicles (EVs), which are a heterogeneous collection of lipid bilayer-enclosed particles released from cells and have the ability to cross the blood-brain barrier (BBB) to deliver functional cargoes to recipient cells 20-22. By incorporating acetylcholine receptor (AChR)-specific rabies virus glycoprotein (RVG) peptides, the EVs were enabled to specifically deliver circSCMH1 to various cell types in the brain 18, 23. Additionally, our previous study indicated that these RVG-circSCMH1-EVs effectively promoted brain repair post-stroke in rhesus monkeys, highlighting their substantial clinical applicability. However, the role of metabolism in the regulatory process of circSCMH1 on brain repair post-stroke remains unknown.

This study investigates the metabolism in the context of circSCMH1-mediated functional recovery following stroke. Our data demonstrated that inhibiting kynurenine 3-monooxygenase (KMO), a metabolic enzyme involved in the kynurenine pathway (KP), improved mitochondrial dynamics and inhibited mitophagy after stroke 24. We further elucidated the specific mechanism by which KMO regulates functional recovery following stroke, underscoring a novel mitochondrial role for KMO. This effect was independent of KMO enzymatic activity, as circSCMH1 did not reduce the 3-hydroxykynurenine (3-HK) concentration in photothrombotic (PT) stroke mice. Mechanically, circSCMH1 bound to STAT5B and inhibited its nuclear translocation, leading to a decrease in KMO expression. Our findings thus revealed that circSCMH1 was able to enhance functional recovery via KMO-regulated mitophagy, providing a previously overlooked mechanism through which circSCMH1 promotes stroke recovery.

## Results

### CircSCMH1 regulates metabolic disorders and inhibits KMO expression after cerebral ischemia

**Figure 1:**
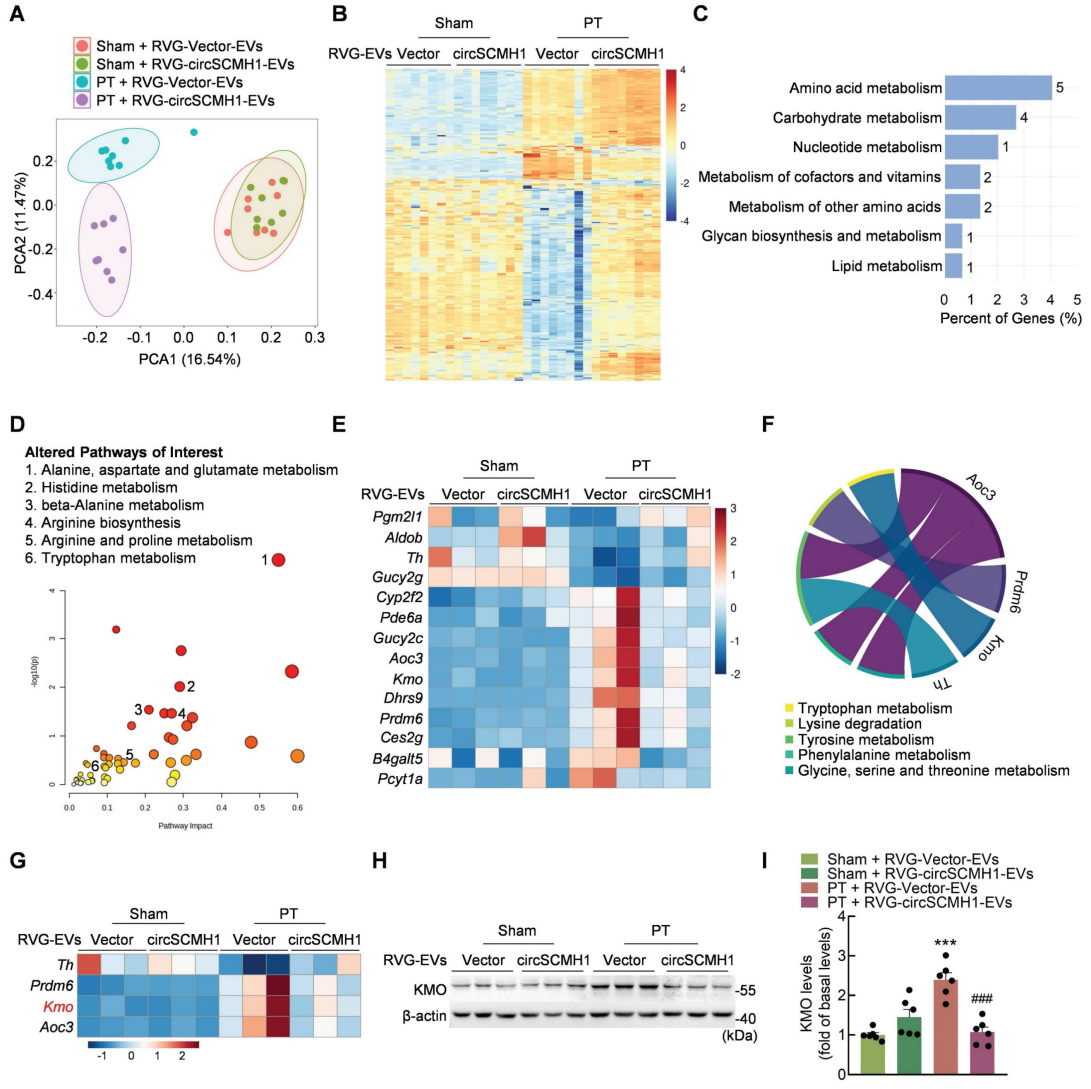
** CircSCMH1 regulates metabolic disorders and inhibits KMO expression after cerebral ischemia.** (**A**) Principal component analysis (PCA) of the metabolome in PT and sham mice overexpressing circSCMH1 or Vector (n = 8). Each symbol represents the data for an individual mouse. (**B**) Heatmap analysis of 346 differential metabolites in PT and sham mice overexpressing circSCMH1 or Vector (n = 8). (**C**) Metabolism-related KEGG pathway enrichment analysis of 148 differentially expressed genes ([log2(fold change)] ≥ 1 and *P*-value < 0.05). (**D**) Pathways altered in PT mice treated with circSCMH1 identified based on transcriptomic and metabolomics data. Metabolic pathways of interest are highlighted. (**E**) Heatmap analysis of 14 genes involved in metabolism identified in transcriptomic analyses (n = 3). (**F**) Chord diagrams visualizing the interrelationships between amino acids metabolism-related genes and amino acids metabolic pathways. (**G**) Heatmap analysis of 4 genes involved in amino acid metabolism identified in transcriptomic analyses (n = 3). (**H, I**) Western blotting analysis of post-stroke KMO expression in mice. Three representative immunoblots are presented from 6 mice/group. ***P < 0.001 versus the sham + RVG-Vector-EVs group; ###*P* < 0.001 versus the PT + RVG-Vector-EVs group using 2-way ANOVA followed by Holm-Sidak post hoc multiple comparisons test. CircSCMH1, circular RNA SCMH1; EVs, extracellular vesicles; KMO, kynurenine 3-monooxygenase; PCA, principal component analysis; PT, photothrombotic stroke; RVG, rabies virus glycoprotein.

Metabolomic analyses were performed on the peri-infarcted cortex of PT mice treated with circSCMH1 on day 3 after stroke. These results revealed significant changes in the metabolomic profile of PT mice compared with sham mice after ischemic stroke, as indicated by principal component analysis (PCA) (Figure 1A). We identified 346 metabolites that changed significantly in PT mice after circSCMH1 treatment that were altered by at least [log2(fold change)] ≥ 1 with a *P*-value < 0.05 (Figure 1B). These significantly altered metabolites were correlated with circSCMH1, and most were associated with amino acids and the amino acid metabolic pathways, as identified using Metaboanalyst (Figure S1A-B). To explore the mechanisms underlying the observed changes in metabolic processes after cerebral ischemia, transcriptomic analyses were performed using samples from the peri-infarcted cortex of PT mice on day 3 post-stroke. A total of 148 genes associated with circSCMH1 were screened (Figure S1C), and subsequent metabolic pathway enrichment analyses revealed a notable enrichment in pathways related to amino acid metabolism (Figure 1C).

Through the integration of RNA-seq and metabolomics data, pathways altered in response to circSCMH1 after PT treatment were identified (Figure 1D), unveiling 14 genes related to metabolism (Figure 1E). As both transcriptomic and metabolomic pathway enrichment analyses revealed the most significant enrichment in amino acid metabolic pathways, four genes (*Th*, *Prdm6*,* Kmo*, and *Aoc3*) that were involved in five amino acid metabolic pathways were screened out (Figure 1F-G). Of these, only *Kmo,* encoding a key enzyme in the KP, exhibited and expression trend that was validated (Figure S1D). RVG-circSCMH1-EVs treatment significantly decreased inducible KMO expression in the peri-infarct cortex of PT mice, as confirmed by western blotting (Figure 1H-I). Taken together, our analyses indicated that circSCMH1 regulated the KP after ischemic stroke by inhibiting the expression of KMO.

### The reparative post-stroke function of circSCMH1 is independent of KMO enzymatic activity

**Figure 2:**
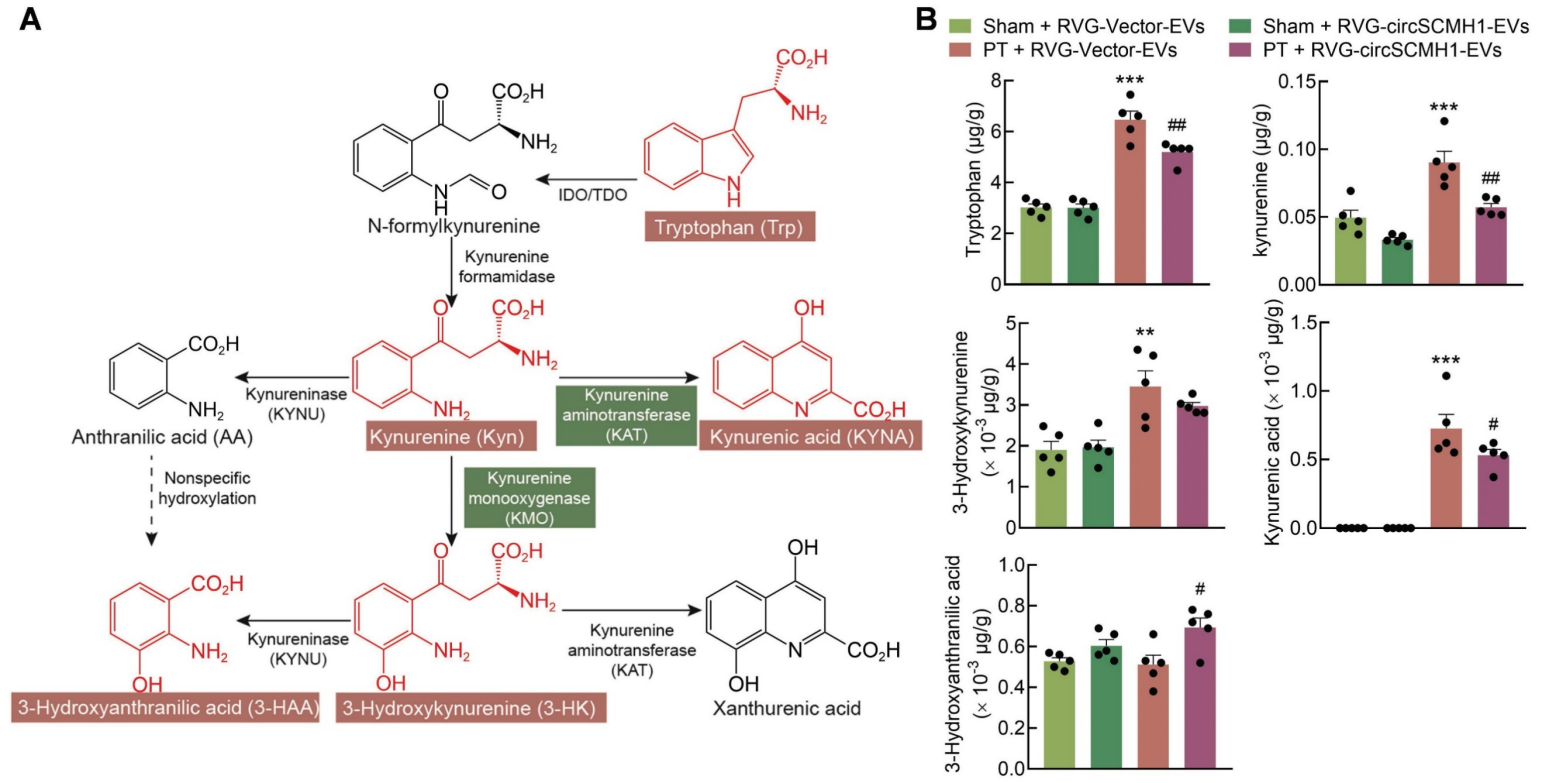
** The reparative post-stroke function of circSCMH1 is independent of KMO enzymatic activity.** (**A**) Diagram of the kynurenine pathway. (**B**) CircSCMH1-treated mouse steady-state kynurenine metabolite tissue concentrations of tryptophan, kynurenine, 3-hydroxykynurenine, kynurenic acid, and 3-hydroxyanthranilic acid. n = 5 animals/group. **P < 0.01, ***P < 0.001 versus Sham + RVG-Vector-EVs group; #*P* < 0.05, ##*P* < 0.01 versus PT + RVG-Vector-EVs group using 2-way ANOVA followed by Holm-Sidak post hoc multiple comparisons test. EVs, extracellular vesicles; KMO, kynurenine 3-monooxygenase; PT, photothrombotic stroke; RVG, rabies virus glycoprotein.

In addition to the observed impact of circSCMH1 on KMO expression, metabolomics analyses revealed that the concentration of tryptophan (Trp) was affected by circSCMH1 treatment. To investigate the effects of circSCMH1 on metabolites involved in the KP, targeted metabolomics analyses were conducted revealing that Trp, kynurenine (Kyn), and 3-HK concentrations increased significantly in the peri-infracted cortex of PT mice. Surprisingly, Trp and Kyn levels decreased significantly following circSCMH1 treatment, while 3-HK levels exhibited a decreasing trend (Figure 2A-B). This result demonstrated that the effect of circSCMH1 on post-stroke functional recovery was independent of the enzymatic activity of KMO, suggesting that they instead act through a novel mechanism. Thus, we hypothesized that circSCMH1 facilitates functional recovery by regulating the expression of KMO.

### KMO inhibition enhances functional recovery after ischemic stroke

**Figure 3:**
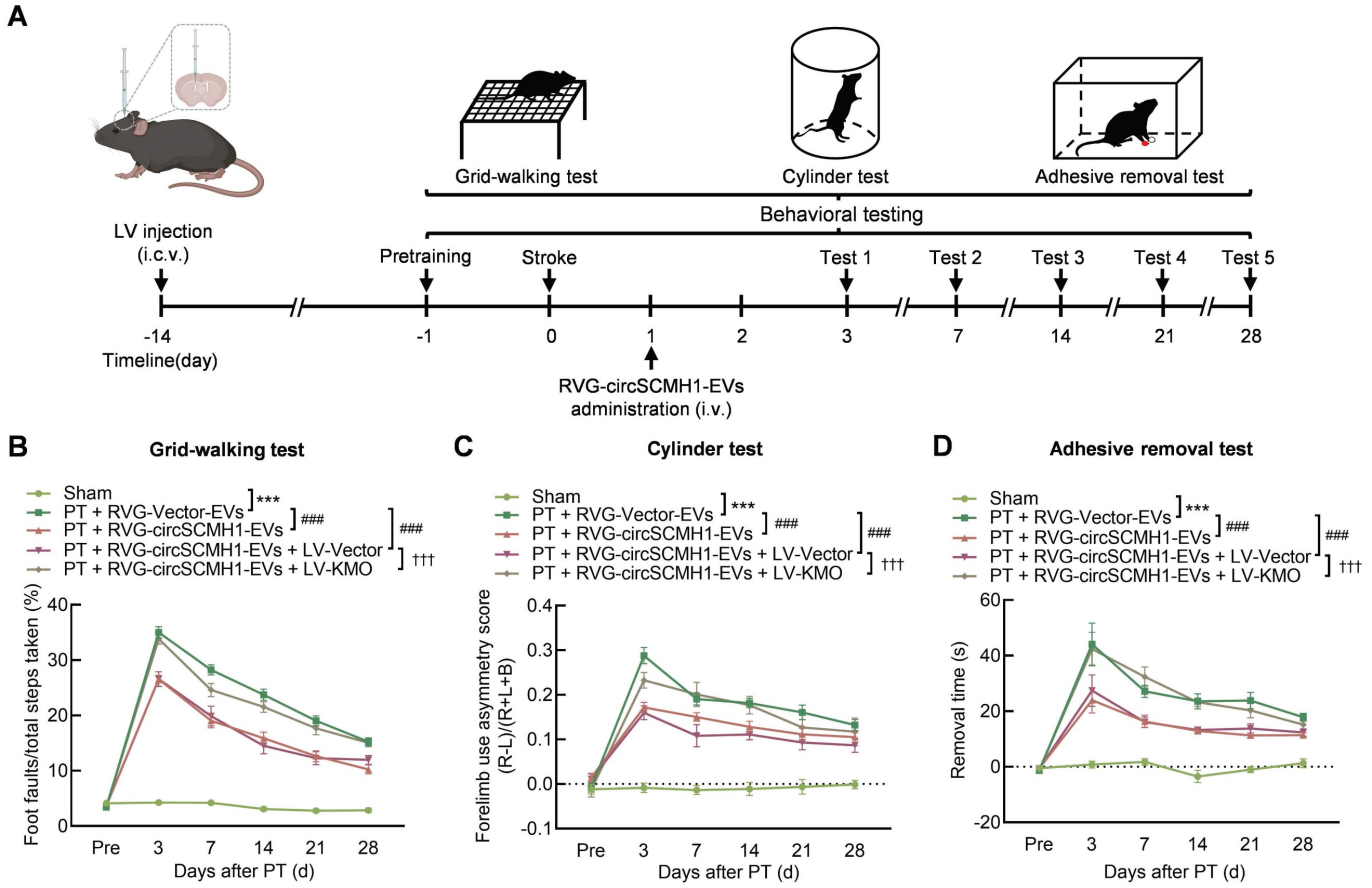
** KMO inhibition enhances functional recovery after ischemic stroke.** (**A**) Schematic overview of LV-KMO, RVG-circSCMH1-EVs administration, and behavioral studies. (**B-D**) KMO eliminated the beneficial effect of circSCMH1 on behavioral recovery at different time points after stroke as measured by the grid-walking test, cylinder test, and adhesive removal test; L indicates left forepaw in the cylinder test; R, right forepaw in the cylinder test; B, both forepaws in the cylinder test. n = 15 animals/group. ***P < 0.001 versus the sham group; ###*P* < 0.001 versus the PT + RVG-Vector-EVs group; †††*P* < 0.001 versus the PT + RVG-circSCMH1-EVs + LV-Vector group using 2-way ANOVA followed by Holm-Sidak post hoc multiple comparisons test. EVs, extracellular vesicles; i.c.v., intracerebroventricular injection; i.v., intravenous injection; KMO, kynurenine 3-monooxygenase; LV, lentivirus; PT, photothrombotic stroke; RVG, rabies virus glycoprotein.

To explore the impact of KMO on the functional recovery of PT mice and to determine whether its effect was regulated by circSCMH1, we overexpressed KMO by microinjecting either the Vector-GFP or KMO-GFP lentivirus (LV) into the contralateral lateral ventricle of mice two weeks before PT surgery. The level of KMO expression was elevated in ipsilateral cortex after KMO-GFP lentivirus microinjecting (Figure S2A-C). Pretrained male mice were subjected to PT followed by intravenous administration of RVG-Vector/circSCMH1-EVs at 24 h after surgery. Behavioral testing was performed from days 3 to 28 after PT stroke (Figure 3A). Treatment with RVG-circSCMH1-EVs at 24 h after stroke promoted motor recovery 18. As shown in behavioral tests, in comparison to the LV-Vector group, animals administered LV-KMO exhibited impaired performance after treatment with RVG-circSCMH1-EVs, as evidenced by increased foot faults on days 3, 7, 14, 21, and 28 following PT surgery (Figure 3B). In the cylinder test, mice treated with LV-KMO showed significantly increased bias at days 3, 7, 14, 21 and 28 after PT model induction (Figure 3C). Furthermore, significant damage-related effects were observed in the adhesive removal test, reflected by the increased removal time after LV-KMO treatment compared with the LV-Vector on days 4, 7, 14, 21, and 28 after PT model induction (Figure 3D). In contrast, LV-Vector treatment did not affect the therapeutic effect of circSCMH1. Collectively, these results demonstrated that circSCMH1 promoted motor recovery by inhibiting the expression of KMO.

### KMO inhibition after circSCMH1 treatment in PT mice regulates mitochondria dynamics

**Figure 4:**
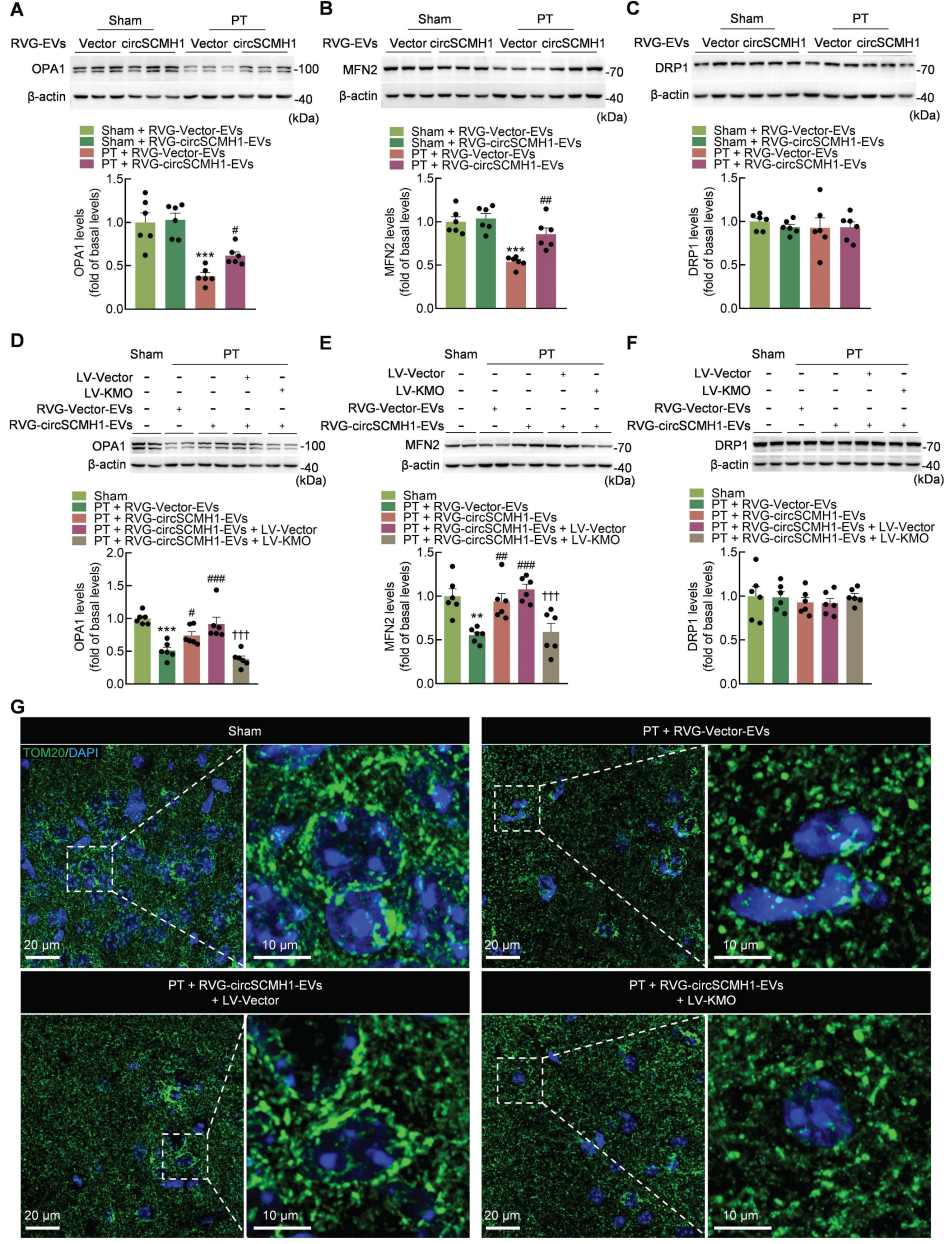
** KMO inhibition after circSCMH1 treatment in PT mice regulates mitochondria dynamics.** (**A-C**) Effect of RVG-circSCMH1-EVs on OPA1, MFN2 and DRP1 levels in mice on day 3 after PT (n = 6 for each group). ***P < 0.001 versus the Sham + RVG-Vector-EVs group; #*P* < 0.05, ##*P* < 0.01 versus the PT + RVG-Vector-EVs group; two-way ANOVA followed by the Holm-Sidak post hoc multiple comparison test. (**D-F**) Representative western blots showing OPA1, MFN2 and DRP1 levels in sham and PT mice after LV-Vector or LV-KMO administration with or without RVG-circSCMH1-EVs treatment (n = 6 for each group). **P < 0.01, ***P < 0.001 versus the Sham group; #*P* < 0.05, ##*P* < 0.01, ###*P* < 0.001 versus the PT + RVG-Vector-EVs group; †††*P* < 0.001 versus the PT + RVG-circSCMH1-EVs + LV-Vector group; two-way ANOVA followed by the Holm-Sidak post hoc multiple comparison test. (**G**) Representative confocal microscopy images of TOM20 (green, a mitochondrial outer membrane marker) in the peri-infarct cortex on day 3 after PT. DAPI, 4′,6-diamidino-2-phenylindole; DRP1, dynamin related protein 1; EVs, extracellular vesicles; KMO, kynurenine 3-monooxygenase; LV, lentivirus; MFN2, mitofusin 2; OPA1, OPA1 mitochondrial dynamin like GTPase; PT, photothrombotic stroke; RVG, rabies virus glycoprotein; TOM20, translocase of outer membrane 20.

The overexpression of human KMO in mammalian cells in a prior study revealed that KMO is involved in the post-translational regulation of DRP1, suggesting that KMO plays a role in mitochondrial dynamics and mitophagy, independent of its enzymatic function in the KP 25. To investigate the effect of circSCMH1 on mitochondrial dynamics through the regulation of KMO expression, its impact on mitochondrial dynamics was examined in PT mice. Treatment with RVG-circSCMH1-EVs significantly increased the levels of OPA1 and MFN2 in the peri-infarct cortex of PT mice, although there was no significant difference in the expression of DRP1 (Figure 4A-C). Meanwhile, KMO overexpression eliminated the increased expression of OPA1 and MFN2 but had no effect on DRP1 expression (Figure 4D-F). Immunofluorescence staining was also used to confirm the effect of circSCMH1 treatment on mitochondrial morphology. This approach revealed that mitochondria exhibited a long tubular shape in sham mice, whereas the mitochondrial network was almost completely fragmented in PT mice. Interestingly, treatment with circSCMH1 significantly inhibited the morphological transition of mitochondria, and this inhibitory effect was abolished by KMO overexpression (Figure 4G). The results showed that mitochondrial dynamics were affected by circSCMH1 through the inhibition of KMO expression.

### CircSCMH1 inhibits mitophagy via suppressing post-stroke KMO expression

**Figure 5:**
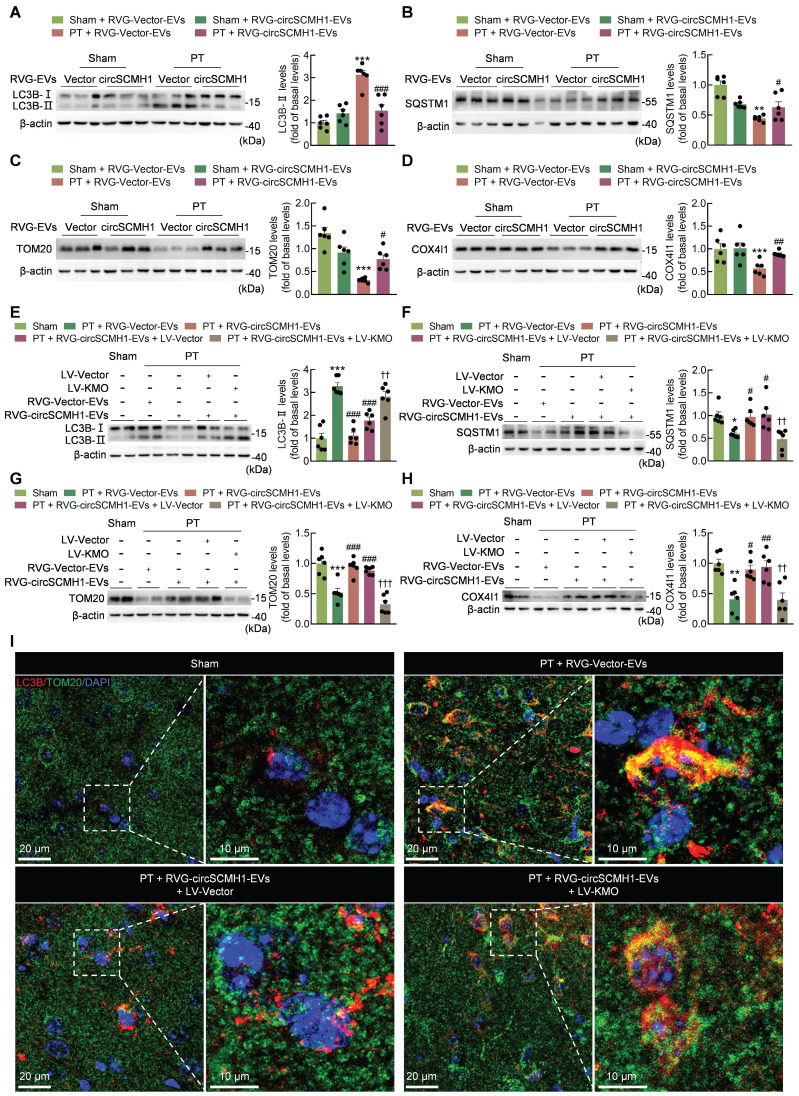
** CircSCMH1 inhibits mitophagy via suppressing post-stroke KMO expression.** (**A-D**) Effect of RVG-circSCMH1-EVs on LC3B, SQSTM1, TOM20, and COX4I1 levels in mice at day 3 after PT (n = 6 for each group). **P < 0.01, ***P < 0.001 versus the Sham + RVG-Vector-EVs group; #*P* < 0.05, ##*P* < 0.01, ###*P* < 0.001 versus the PT + RVG-Vector-EVs group using two-way ANOVA followed by the Holm-Sidak post hoc multiple comparison test. (**E-H**) Representative western blots of LC3B, SQSTM1, TOM20 and COX4I1 levels after LV-Vector or LV-KMO administration with or without RVG-circSCMH1-EVs treatment (n = 6 for each group). *P < 0.05, **P < 0.01, ***P < 0.001 versus the Sham group; #*P* < 0.05, ##*P* < 0.01, ###*P* < 0.001 versus the PT + RVG-Vector-EVs group; ††*P* < 0.01, †††*P* < 0.001 versus the PT + RVG-circSCMH1-EVs + LV-Vector group using two-way ANOVA followed by the Holm-Sidak post hoc multiple comparison test. (**I**) Representative confocal microscopy images of TOM20 (green, a mitochondrial outer membrane marker) and LC3B in the peri-infarct cortex at day 3 after PT. COX4I1, cytochrome c oxidase subunit 4I1; DAPI, 4′,6-diamidino-2-phenylindole; EVs, extracellular vesicles; KMO, kynurenine 3-monooxygenase; LC3B, microtubule associated protein 1 light chain 3 beta; LV, lentivirus; PT, photothrombotic stroke; RVG, rabies virus glycoprotein; SQSTM1, sequestosome 1; TOM20, translocase of outer membrane 20.

Mitochondrial fusion/fission induce mitophagy 26. In order to explore the effect of circSCMH1 on mitophagy, the levels of proteins that associated with mitophagy activation were examined. We found that circSCMH1 treatment led to a decrease in the expression of LC3B (microtubule-associated protein 1 light chain 3 beta)-II, a general autophagosomal marker, in PT mice (Figure 5A), indicating that circSCMH1 prevented the formation of autophagosomes. This conclusion was supported by the results of SQSTM1 (sequestosome 1; a receptor protein that links LC3B with ubiquitin) immunoblotting performed on PT mice after circSCMH1 therapy (Figure 5B). Furthermore, the levels of the mitochondrial proteins TOM20 and COX4I1, which are indicators of mitochondrial content, decreased in the brains of PT-treated mice, while circSCMH1 treatment attenuated the decrease in these proteins (Figure 5C-D).

Next, we investigated whether circSCMH1 was able to affect mitophagy by regulating the expression of KMO. The PT mice overexpressing KMO exhibited a dramatic reversal of the elevated levels of the mitochondrial marker proteins (TOM20 and COX4I1), together with the elevation of LC3B-II and a reduction in the autophagy substrate SQSTM1 in response to circSCMH1 therapy (Figure 5E-H). These results indicated that KMO alleviated the inhibitory effect of circSCMH1 on mitophagy. To further confirm the effect of circSCMH1 on mitophagy, co-staining for LC3B and TOM20 was performed. In the peri-infarct cortex of PT mice, the notable co-localization of TOM20 and LC3B was observed, indicative of an increased level of mitophagy. Notably, treatment with circSCMH1 resulted in a reduction in their co-localization, suggesting an inhibitory effect of circSCMH1 on mitophagy. Additionally, the overexpression of KMO led to an increase in the co-localization of TOM20 and LC3B, implicating KMO in the regulation of mitophagy (Figure 5I). These results supported the conclusion that circSCMH1 inhibited mitophagy by suppressing KMO expression after PT treatment.

### Glial activation is reduced by the inhibition of KMO expression

In the brain, KMO exerts its functions in various cells and does not exhibit any clear cell type specificity 27-31. Using markers for neurons (NeuN^+^), astrocytes (GFAP^+^ [glial fibrillary acidic protein^+^]), microglia (Iba-1^+^), and endothelial cells (CD31^+^), serial sections of the lesioned brains of PT mice were processed for immunofluorescence staining to determine the cellular distribution of KMO in the peri-infarct cortex. Neurons, astrocytes, microglia, and endothelial cells all colocalized with the KMO signal in the peri-infarct cortex (Figure S3). These results indicated that KMO had no significant cell specificity in the brain.

**Figure 6:**
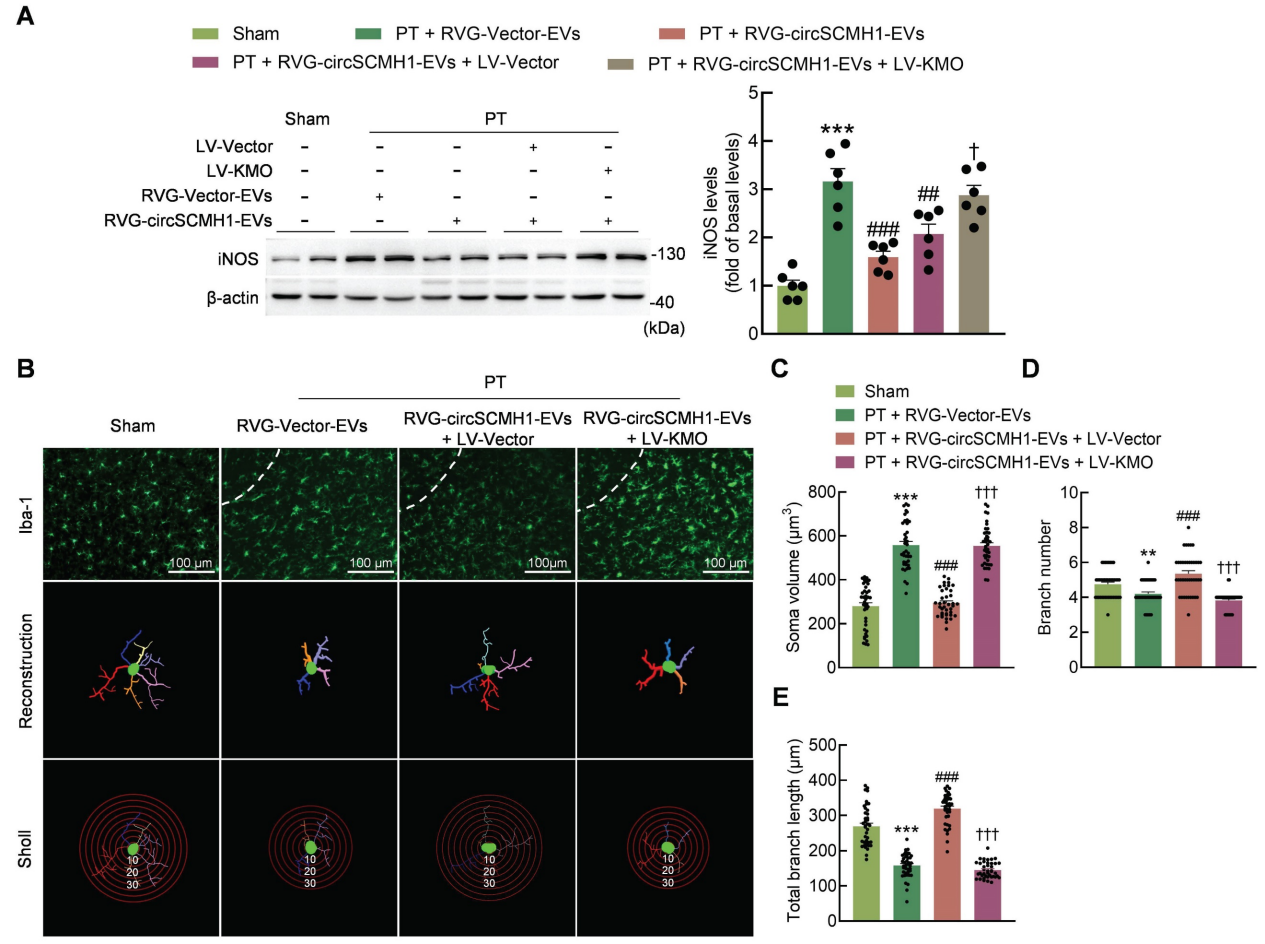
** Microglial activation is reduced by the inhibition of KMO expression.** (**A**) Western blotting analysis of iNOS expression after LV-KMO and RVG-circSCMH1-EVs treatment in mice at day 3 after PT. Two representative immunoblots are presented from 6 mice/group. ***P < 0.001 versus the Sham group; ##*P* < 0.01, ###*P* < 0.001 versus the PT + RVG-Vector-EVs group; †*P* < 0.05 versus the PT + RVG-circSCMH1-EVs + LV-Vector group using two-way ANOVA followed by the Holm-Sidak post hoc multiple comparison test. (**B-E**) KMO abolished the inhibitory effect of RVG-circSCMH1-EVs on microglial reactivity in the peri-infarcted cortex on day 3 after PT in mice. Representative images of microglial immunostaining for Iba-1, followed by 3D reconstruction and Sholl analysis (**B**). Average soma volume (**C**), branch number (**D**), total branch length (**E**) (n = 4 mice/group, 40 cells/group). **P < 0.01, ***P < 0.001 versus the Sham group; ###*P* < 0.001 versus the PT + RVG-Vector-EVs group; †††*P* < 0.001 versus the PT + RVG-circSCMH1-EVs + LV-Vector group; two-way ANOVA followed by the Holm-Sidak post hoc multiple comparison test. EVs, extracellular vesicles; iNOS, inducible nitric oxide synthase; KMO, kynurenine 3-monooxygenase; LV, lentivirus; PT, photothrombotic stroke; RVG, rabies virus glycoprotein.

**Figure 7:**
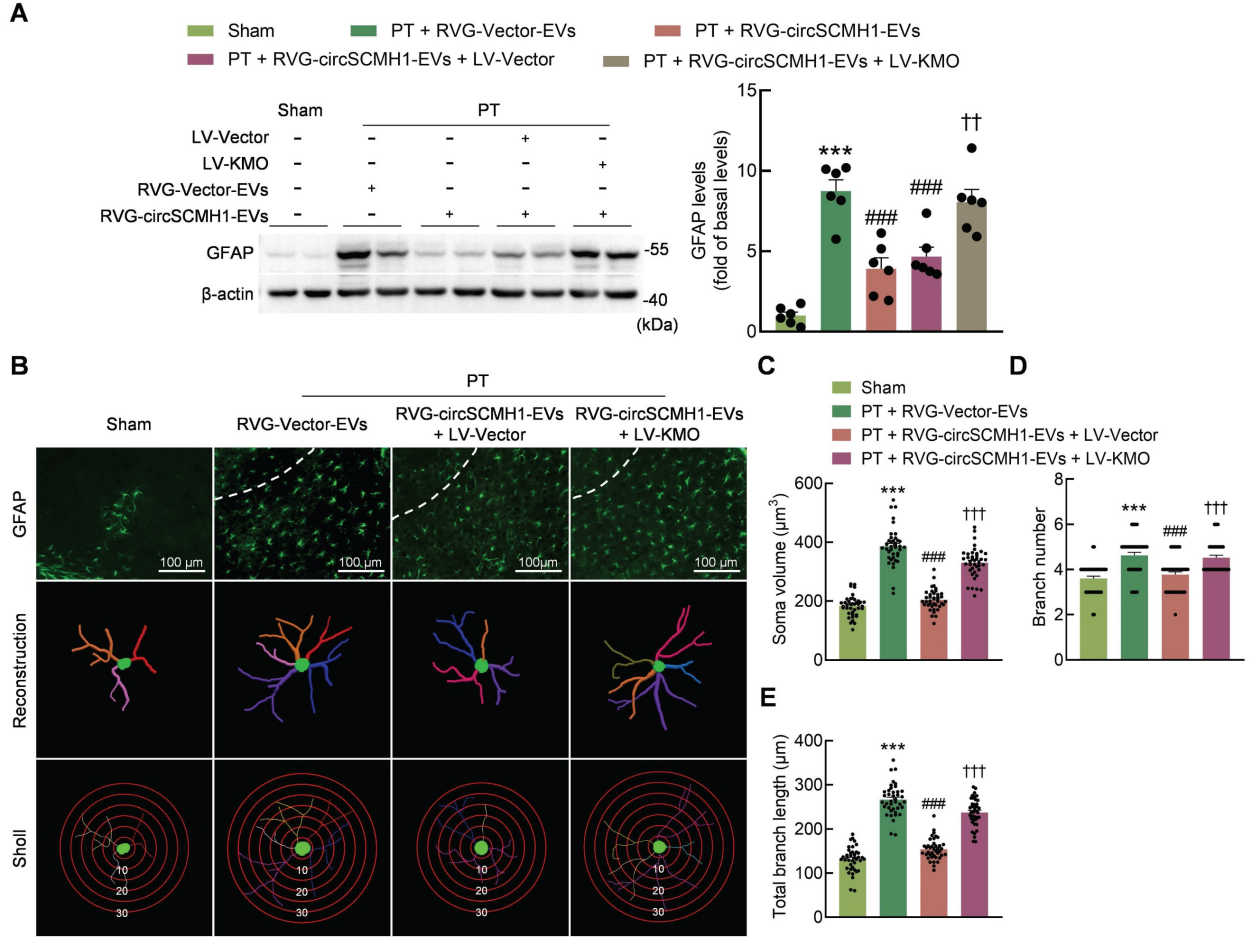
** Astrocyte activation is reduced by the inhibition of KMO expression.** (**A**) Western blot analysis of GFAP expression after LV-KMO and RVG-circSCMH1-EVs treatment in mice at day 3 after PT. Two representative immunoblots were presented from 6 mice/group. ***P < 0.001 versus the Sham group; ###*P* < 0.001 versus the PT + RVG-Vector-EVs group; ††*P* < 0.01 versus the PT + RVG-circSCMH1-EVs + LV-Vector group using two-way ANOVA followed by the Holm-Sidak post hoc multiple comparison test. (**B-E**) KMO abolished the inhibitory effect of RVG-circSCMH1-EVs on astrocyte reactivity in the peri-infarcted cortex on day 3 after PT in mice. Representative images of astrocyte immunostaining for GFAP, followed by 3D reconstruction and Sholl analysis (**B**). Average soma volume (**C**), branch number (**D**), total branch length (**E**) (n = 4 mice for each group, 40 cells for each group). ***P < 0.001 versus the Sham group; ###*P* < 0.001 versus the PT + RVG-Vector-EVs group; †††*P* < 0.001 versus the PT + RVG-circSCMH1-EVs + LV-Vector group using two-way ANOVA followed by the Holm-Sidak post hoc multiple comparison test. EVs, extracellular vesicles; GFAP, glial fibrillary acidic protein; KMO, kynurenine 3-monooxygenase; LV, lentivirus; PT, photothrombotic stroke; RVG, rabies virus glycoprotein.

CircSCMH1 significantly reduces astrocytic and microglial reactivity after cerebral ischemia 18, although the mechanism is still unclear. Western blotting showed that KMO overexpression eliminated the inhibitory effect of circSCMH1 on iNOS (Figure 6A). This finding was confirmed by immunostaining, as KMO overexpression eliminated the inhibitory effect of circSCMH1 on microglial activation by increasing microglial soma size and branch number, while decreasing branch length in PT mice (Figure 6B-E). The same conclusion was drawn in astrocytes, as the overexpression of KMO promoted astrocyte activation after circSCMH1 treatment, as characterized by significantly increased GFAP levels (Figure 7A). Meanwhile, immunostaining revealed significantly increased soma size, branch number, and branch length (Figure 7B-E). These results indicated that circSCMH1 regulates mitophagy through KMO, thereby impacting glial cell activation.

### Neuroplasticity and vascular remodeling are enhanced by the inhibition of KMO expression

In addition to affecting glial activation, circSCMH1 also improves neuroplasticity and vascular remodeling after ischemic stroke 18, 19. Western blotting was used to examine the protein levels of PSD95 and synaptophysin, and the results showed that the PT mice overexpression KMO exhibited a dramatic reversal of the upregulation of the neuroplasticity markers PSD95 and synaptophysin induced by circSCMH1 treatment (Figure S4A-B). Furthermore, the fluorescence intensity of MAP2 was significantly increased in circSCMH1-treated mice and KMO overexpression alleviated this effect (Figure S4C-D).

To investigate the potential involvement of KMO in vascular repair induced by circSCMH1 during ischemic stroke, KMO was overexpressed via lentiviral microinjection. On day 28 after PT model induction, KMO overexpression markedly reversed the elevated levels of tight junction proteins (ZO-1 and Occludin) induced by circSCMH1 treatment (Figure S5A-B). Additionally, immunostaining demonstrated that KMO overexpression significantly decreased vascular area, and total vascular length in PT mice treated with circSCMH1 (Figure S5C-E). These results indicated that KMO overexpression eliminated the beneficial effects of circSCMH1 on neuroplasticity and vascular remodeling after cerebral ischemia.

### CircSCMH1 decreases *Kmo* expression and binds to STAT5B

**Figure 8:**
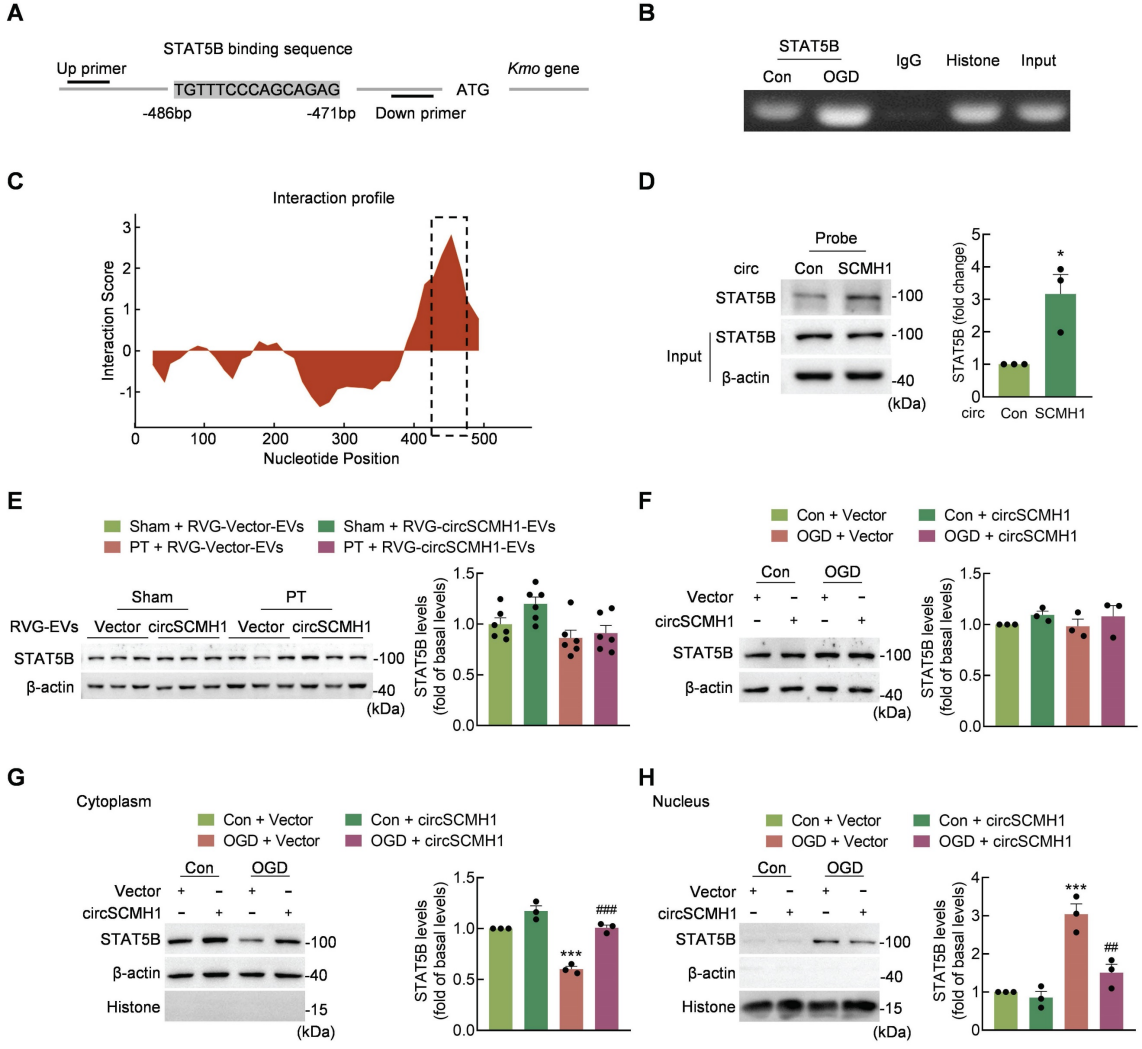
** CircSCMH1 decreases *Kmo* expression and binds to STAT5B.** (**A**) Illustration of the consensus binding site between STAT5B and the *Kmo* promoter. (**B**) ChIP assay demonstrating the ability of STAT5B to bind to *Kmo* promoter. (**C**) Prediction of circSCMH1-STAT5B interaction using the catPAPID algorithm. (**D**) Interaction between circSCMH1 and STAT5B was measured by RNA pull-down assay in HT-22 cells. All data were presented as mean ± SEM of three independent experiments. *P < 0.05 versus the circControl group using Student's t-test. (**E**) Representative western blotting analysis of STAT5B levels after RVG-circSCMH1-EVs administration with or without PT treatment (n = 6 for each group). The significance was analyzed using two-way ANOVA followed by the Holm-Sidak post hoc multiple comparison test. (**F-H**) Western blotting analysis of STAT5B protein levels (**F**) and cytoplasmic (**G**) or nuclear (**H**) localization of STAT5B in HT-22 cells after vector or circSCMH1 plasmid transduction with or without OGD treatment for 3 h. All data are presented as means ± SEM of three independent experiments. ***P < 0.001 versus the Control + Vector group; ##*P* < 0.01, ###*P* < 0.001 versus the OGD + Vector group; two-way ANOVA followed by the Holm-Sidak post hoc multiple comparison test. Con, control; EVs, extracellular vesicles; OGD, oxygen glucose deprivation; RVG, rabies virus glycoprotein; STAT5B, signal transducer and activator of transcription 5B.

To identify the molecular mechanisms involved in the regulation of *Kmo* expression by circSCMH1, the transcription factors predicted to regulate *Kmo* were identified using the JASPAR, AnimalTFDB and ALGGEN, databases pertaining to human and mouse transcriptional regulation. Based on the intersection of these three databases, we selected seven transcription factors that bound to the sense strand of *Kmo*. We then used the catRAPID algorithm to predict the interaction between circSCMH1 and transcription factors, revealing that STAT5B bound circSCMH1 most significantly (Table S1). In addition, ChIP experiments showed that oxygen glucose deprivation (OGD) treatment of HT-22 cells increased STAT5B binding to the *Kmo* promoter (Figure 8A-B). Meanwhile, one region of circSCMH1 (positions 426-485) exhibited a high capacity for interaction with STAT5B, and RNA pull-down assays using a biotinylated circSCMH1 probe revealed that STAT5B was pulled down at higher levels by the circSCMH1 probe as compared with the circControl probe (Figure 8C-D).

We next explored how circSCMH1 was able to regulate STAT5B as a means of affecting *Kmo* expression. Western blotting demonstrated that treatment with circSCMH1 did not alter STAT5B expression in PT mice (Figure 8E). Similarly, the total expression of STAT5B in HT-22 cells following OGD treatment did not differ significantly between circSCMH1-overexpressed and circControl groups (Figure 8F). Notably, after OGD treatment, circSCMH1 decreased the level of STAT5B in the nucleus and increased it in the cytoplasm of HT-22 cells (Figure 8G-H). Based on these results, we concluded that circSCMH1 regulates *Kmo* expression by binding to STAT5B and inhibiting its translocation to the nucleus.

## Discussion

In this study, we explored the mechanism by which KMO is involved in circSCMH1-mediated functional recovery after ischemic stroke. CircSCMH1 was found to promote functional recovery after stroke by regulating KMO expression, which was independent of KMO enzymatic activity. Mechanistically, circSCMH1 decreased the nuclear translocation of STAT5B via direct binding to STAT5B, resulting in decreased *Kmo* expression, and thereby promoting mitochondrial fusion and inhibiting mitophagy. Finally, microglial and astrocyte activation were inhibited and neural plasticity and vascular remodeling were promoted by this circRNA, ultimately improving post-stroke repair.

Metabolic and ionic disturbances cause various forms of downstream injury after cerebral ischemia 32. In this study, coordinated metabolomic and RNA-seq analyses were used to identify the metabolic pathways involved in ischemic stroke, revealing that the KP was regulated by circSCMH1.

In recent years, aberrant activation of the KP has been reported to be highly associated with ischemic stroke. Plasma levels of Trp hydroxylase and 3-HAA are downregulated in acute ischemic stroke (AIS) patients 33, 34, and an elevated serum quinolinic acid/kynurenic acid (QUIN/KYNA) ratio is a reliable biomarker of cognitive decline following stroke 35. However, it is unclear how KP participates in the pathologic processes of ischemic stroke.

Transcriptomic analyses revealed that the level of *Kmo* was significantly increased after cerebral ischemia, and its expression was regulated by circSCMH1. Inhibition of KMO should reduce 3-HK production, and may therefore provide an efficacious approach to preventing or reducing the severity of ischemic stroke 36, 37. However, we found that treatment with circSCMH1 did not affect the concentration of 3-HK after stroke, suggesting that the post-stroke repair function of circSCMH1 was independent of KMO catalytic activity. KMO is a mitochondrial protein that localizes to the outer mitochondrial membrane in eukaryotic cells, and overexpression of KMO affects mitochondrial dynamics 25. We found that the overexpression of KMO eliminated the beneficial effects of circSCMH1 on mitophagy and functional recovery after ischemic stroke. Together, our results indicated that circSCMH1 plays a role in post-stroke repair by inhibiting KMO-mediated mitophagy.

Our previous research indicates that circSCMH1 is a viable therapeutic target 18, 19. Mitophagy regulates glial activation 38-40, which is a promising target for efforts to control cerebral ischemic injury. In the current study, the inhibitory effect of circSCMH1 on microglial and astrocyte activation was abrogated by KMO overexpression in the peri-infarct cortical areas of mice after PT stroke. Moreover, circSCMH1 affected neuroplasticity and vascular repair after stroke through the regulation of KMO expression. Together, our results indicated that circSCMH1-mediated decreases in KMO expression inhibited glial activation following ischemic stroke.

Increasing evidence indicates that circRNAs exhibit diverse biological functions 41. Our previous experiments have demonstrated that circSCMH1 binds to MeCP2 18. In addition to serving as a "miRNA sponge", circRNAs engage with RNA-binding proteins and influence their distribution and function, as supported by recent research 42, 43. In the present study, we found that circSCMH1 inhibited the expression of *Kmo* by binding to the transcriptional activator STAT5B 44, resulting in the inhibition of STAT5B in the nucleus and decreased expression of the downstream molecule KMO. Thus, circSCMH1 appears to have multi-target effects, suggesting that it is a promising therapeutic candidate for the treatment of ischemic stroke.

Using the ALGGEN, JASPAR, and AnimalTFDB algorithms, STAT5B binding sites located within the promoter of *Kmo* were predicted. Consistent with these predictions, STAT5B was herein found to directly regulate *Kmo* expression in Chromatin immunoprecipitation (ChIP) assays. Additionally, bioinformatic prediction and pull-down assays were performed to confirm the interaction between STAT5B and circSCMH1. STAT5B is a member of the STAT protein family, members of which are transcription factors involved in various cellular processes including cell growth, differentiation, and apoptosis 45. Previous studies have demonstrated that rats with localized cerebral ischemia and reperfusion exhibit markedly altered STAT5b levels in the hippocampus 46. Our results suggest that STAT5B regulates the expression of KMO, with this activity in turn being regulated by circSCMH1.

The majority of global stroke guidelines now recommend intravenous thrombolysis with alteplase within 4.5 h of symptom onset 47. Additionally, endovascular thrombectomy extends the therapeutic window for patients up to 12 h after stroke onset by rapidly reopening occluded vessels and reestablishing tissue perfusion 21. In recent years, advancements in targeted delivery technologies and improvements in the stability and safety of nucleic acid drugs have made them a focal point in various fields of disease research. Our previous studies have shown that circSCMH1-based therapies have a relatively wider potential therapeutic window. Experimental results in non-human primate models demonstrated that RVG-circSCMH1-EVs administered 24 h after stroke significantly facilitate post-stroke brain repair, providing a more practical therapeutic window compared to existing treatments, and carrying substantial clinical implications 18. This current study elucidated the specific mechanisms by which circSCMH1 improved functional repair after stroke by regulating mitophagy, thereby reinforcing the potential of circSCMH1 as a promising clinical therapeutic target for stroke. Our findings provide a solid theoretical foundation for advancing clinical translational research in circSCMH1.

Our study has several limitations. First, although our results demonstrating enhanced post-stroke benefits are promising, a larger sample size would increase statistical power. This expansion could potentially reveal additional significant relationships or effects undetected in our current study. Second, our study may be susceptible to potential biases arising from inherent methodological constraints. For example, the results of targeted metabolomics are particularly vulnerable to variations in sample processing procedures, which could introduce unintended confounding factors. Third, while our results are promising, further validation in clinical settings is necessary before drawing definitive conclusions or implementing changes in clinical practice.

Taken together, the findings of this study highlight the functional link between KMO and functional recovery after stroke: The overexpression of KMO was able to promote mitophagy and aggravate post-stroke injury. circSCMH1 was found to bind to STAT5B and interfere with its nuclear translocation, leading to decreased expression of KMO, with subsequent improvements in motor functional behaviors. Our study thus provides a proof-of-concept that circSCMH1 can function as a novel inhibitor of KMO and thus has the potential to be a therapeutic target for efforts to manage metabolic disorders after cerebral ischemia.

## Materials and Methods

### EV isolation and purification

HEK293T (Chinese Academy of Sciences, Shanghai, China) cells were grown in 5% CO_2_ in Dulbecco's modified Eagle's medium (DMEM, 10-013-CVR, Corning), supplemented with dialyzed fetal bovine serum (10% v/v, 26400044, Gibco). When the cells reached approximately 70%-80% confluence, they were co-transfected with the vector or circSCMH1-GFP plasmid and RVG-Lamp2b plasmid (71294, ADDGENE) using Lipofectamine 2000 (11668019, Invitrogen) according to the manufacturer's instructions. Forty-eight hours after transfection, hygromycin B (100 μg/ml, 108435550, Roche) and puromycin (200 ng/ml, AK058, GPC Biotechnology) were added to the cell culture medium to obtain stable strains. EVs were isolated from cell culture supernatants. Conditional medium was centrifuged at 300 × *g* for 5 min, 3,000 × *g* for 15 min, and 10,000 × *g* for 60 min at 4 ℃ to remove cells and debris. The supernatants were filtered with 0.22 μm pore filters and centrifuged at 200,000 × *g* for 90 min at 4 ℃. Finally, the pellet was resuspended with PBS followed by another centrifugation step at 200,000 × *g* to purify EVs.

### Animals

We conducted our tests using an all-male animal colony to eliminate the disruption of female physiological cycles and reduce the influence elements of the experiment since male and female mice differ physiologically, especially in the production of hormones. Adult male C57BL/6J mice (24.0-26.0 g, 8-10 weeks old) were purchased from the GemPharmatech (Nanjing, China) and randomly assigned to experimental groups. All of the animals were kept in climate-controlled environments with consistent humidity and a 12-hour light/12-hour dark cycle, with the lights turned on at 7:00 AM. Food and water were available *ad libitum*. All animal experiments were approved by the Institutional Animal Care and Use Committee (IACUC) at the Medical School of Southeast University (approval ID: 20210325067) and performed in accordance with the Animal Research: Reporting of *In Vivo* Experiments (ARRIVE) guidelines. Every attempt was made to use as few animals as possible. Total 285 mice were included in this study, and 6 mice were excluded due to death (n = 2) or failed stroke induction (n = 4). Sample size required for animal study was empirically based upon the results of previous experiments and similar to that generally used in the field. Mice were coded and randomly divided into experimental and control groups.

### Photothrombotic (PT) stroke mouse model

As previously described 48, 49, focal cortical ischemia was induced in mice by photothrombosis of cortical microvessels. Briefly, mice were anesthetized in a closed anesthesia chamber containing 3% isoflurane (R510-22, RWD), 30% oxygen, and 70% carbon dioxide. The rectal temperature was controlled at 37.0 ± 0.5 °C with a homeothermic blanket during surgery. Mice were treated with Rose Bengal (30 mg/kg, i.v., 330000, Sigma) and then placed in a stereotaxic device. Cut the skin from the midline to expose the skull, and then cleared the surface. A cold light source (World Precision Instruments, USA) attached to an opaque template with a 2 mm diameter was positioned 1.5 mm lateral from the bregma. Five minutes after Rose Bengal administration, the brain was illuminated for 5 min, the illumination is 12,000 lux. With light excitation, singlet oxygen was generated from Rose Bengal, which damaged and occluded vascular endothelium. Sham mice received the same dose of Rose Bengal without illumination.

### Intravenous delivery of circSCMH1 in animals

RVG-circSCMH1-EVs or RVG-Vector-EVs (12 mg/kg) were administered intravenously. To evaluate the effect of circSCMH1 on KMO expression and mitochondria dynamics in the PT stroke model, we divided the mice into the following groups: Sham + RVG-Vector-EVs, Sham + RVG-circSCMH1-EVs, PT + RVG-Vector-EVs, and PT + RVG-circSCMH1-EVs (n = 6 animals/group).

To explore the effect of KMO on functional recovery after stroke, we formed the following groups: Sham, PT + RVG-Vector-EVs, PT + RVG-circSCMH1-EVs, PT + RVG-circSCMH1-EVs + LV-Vector, PT + RVG-circSCMH1-EVs + LV-KMO (n = 15 animals/group).

To confirm the function of circSCMH1 was exerted by regulating KMO expression, we formed the following groups: Sham, PT + RVG-Vector-EVs, PT + RVG-circSCMH1-EVs, PT + RVG-circSCMH1-EVs + LV-Vector, PT + RVG-circSCMH1-EVs + LV-KMO (n = 6 animals/group).

### RNA-sequencing analysis

Whole RNA-sequencing analysis was performed by LC-Bio (Hangzhou, China). We divided the mice into the following groups: Sham + RVG-Vector-EVs, Sham + RVG-circSCMH1-EVs, PT + RVG-Vector-EVs, and PT + RVG-circSCMH1-EVs (n = 3 animals/group). RVG-circSCMH1-EVs or RVG-Vector-EVs (12 mg/kg) were administered intravenously 24 h after PT molding. The peri-infarct cortex from these mice were collected in TRIzol. Total RNAs were extracted from TRIzol. Each sequence fragment was given a sequence tag using UMI technology, which minimizes the interference of duplication generated by PCR amplification on the quantitative accuracy of transcriptome. Using Hisat2 (2.0.4), RNA-seq data were aligned to the mouse genome (GRCh37/hg19). Fragments per kilobase of exon per million fragments mapped (FPKM) was used to measure transcript abundance. DESeq2 was used to identify the genes that were expressed differently 50. The Gene Expression Omnibus (GEO) accession code for the data in this manuscript is GSE261613.

### Metabolomics analysis

The collected samples were thawed on ice, and metabolite were extracted with 80% methanol buffer. Briefly, 50 mg of sample was extracted with 0.5 ml of precooled 80% methanol. The extraction mixture was then stored in 30 min at -20 °C. After centrifugation at 20,000 × *g* for 15 min, the supernatants were transferred into new tube to and vacuum dried. The samples were redissolved with 100 μl 80% methanol and stored at -80 °C prior to the LC-MS analysis.

All samples were acquired by the LC-MS system followed machine orders. Firstly, all chromatographic separations were performed using an UltiMate 3000 UPLC System (Thermo Fisher Scientific, Bremen, Germany). An ACQUITY UPLC T3 column (100 mm × 2.1 mm, 1.8 µm, Waters, Milford, USA) was used for the reversed phase separation. The column oven was maintained at 35 °C. It was introduced for the separation of metabolites that the mobile phase consisted of solvent A (water, 0.1% formic acid) and solvent B (Acetonitrile, 0.1% formic acid). The gradient elution conditions were as follows with a flow rate of 0.4 ml/min: 5% solvent B for 0-0.5 min; 5-100% solvent B for 0.5-7 min; 100% solvent B for 7-8 min; 100-5% solvent B for 8-8.1 min; and 5% solvent B for 8.1-10 min. A high-resolution tandem mass spectrometer Q-Exactive (Thermo Scientific) operating in positive and negative ion modes was used to scan from m/z 70-1,000 at 70,000 resolution.

The acquired MS data pretreatments including peak picking, peak grouping, retention time correction, second peak grouping, and annotation of isotopes and adducts was performed using XCMS software. The online KEGG, HMDB database was used to annotate the metabolites by matching the exact molecular mass data (m/z) of samples with those from database. The intensity of peak data was further preprocessed by metaX. Student t-tests were conducted to detect differences in metabolite concentrations between 2 phenotypes (*P* < 0.05).

### Liquid chromatography-tandem mass spectrometry (UHPLC-MS/MS) analysis of tryptophan metabolites

Tissues were fragmented in fivefold volume of ice-cold methanol. The samples were incubated for 20 min at -20 ℃. The supernatant was collected by centrifugation, and the precipitate was extracted in duplicate. The supernatant was pooled and dried under nitrogen. Samples were redissolved with 50 μl of 10% aqueous methanol containing 250 ng/ml of Tryptophan-^13^C_11_ internal standard. A standard mixture was newly prepared from single standard stock solution. The standard mixture was serially diluted to appropriate concentration ranges. The calibration curve sample were obtained by mixing 50 μl of the above standard mixture with 12.5 ng tryptophan-^13^C_11_ as internal standard in insert vials, respectively. Ten-microliter volumes of each sample were injected onto a Waters ACQUITY UPLC HSS T3 Column (1.8 µm; 100 × 2.1 mm internal diameter, Waters, Elstree, Herts., UK) using an Agilent UPLC, coupled to an Agilent 6470 mass spectrometer. The flow rate was 0.25 ml/min. Separation was carried out using an A-water, B-acetonitrile (both containing 0.1% formic acid). The chromatographic separation was conducted by a gradient elution program as follows: 0-0.5 min, 1% B; 7 min, 60% B; 7.5 min, 99% B; 9 min, 99% B; 9.1 min, 1% B; 11 min, 1% B. The mass spectrometer was operated in positive ion electrospray mode. The Agilent MassHunter software (version B.08.00) was used to control instruments and acquire data.

### Mouse behavioral tests

The mice were coded and were randomly divided into five groups. Mouse behavioral tests were performed by an independent investigator who was blind to the experimental group.

For the grid-walking task, an elevated grid area of 32 cm × 20 cm × 50 cm (length × width × height) made of 12 mm square wire mesh was used 48, 51. Each mouse was placed separately on the wire grid and was free to wander about until the left forelimb had taken at least 100 steps. Under the grid, a camera was placed to capture stepping errors (foot faults). In brief, it was determined to be a foot fault: 1) if a step was unable to support the foot, causing the foot to go through the grid hole; 2) if the grid was level with the mouse's wrist and it was at rest. The numbers of foot faults and non-faults for each limb were counted. A ratio was calculated as follows: number of foot faults / (number of foot faults + number of non-faults) × 100%.

For the cylinder test 52, mice were put within a 10 cm in diameter by 15 cm height plastic cylinder, and videotaping was done for 5 min. The score was calculated as the ratio: (number of right hand - number of left hand) / (number of right hand + number of left hand + number of both hands).

For the adhesive removal somatosensory test 16, 53, 2 small pieces of adhesive-backed paper dots (of equal size, 25 mm^2^) were used as bilateral tactile stimuli occupying the distal-radial region on the wrist of each forelimb. Three trials per day were used to time how long it took the mice to remove each stimulus from the forelimb. Individual trials were separated by at least 5 min. Animals had three days of training prior to surgery. Once mice were able to remove the dots within 10 s, they were subjected to stroke. The result was calculated as follows (time in second): time of left hand-time of right hand.

### Immunostaining and image analysis of sections

With the help of a cryostat, sections were divided into 30 μm coronal slices. After that, they were incubated in 0.3% Triton X-100 in PBS for 15 min, followed by blocking in 10% normal goat serum (ZLI-9056, ZSGB-BIO) in 0.3% Triton X-100 for an hour at room temperature. Next, the sections were incubated with a rabbit anti-TOM20 antibody (1:100, 11802-1-AP, Proteintech), mouse anti-LC3B antibody (1:100, MA5-37852, Invitrogen), rabbit anti-Iba-1 antibody (1:200, 019-19741, Wako), mouse anti-GFAP antibody (1:200, G3893-.2ML, sigma), rabbit anti-KMO antibody (1:50, 10698-1-AP, Proteintech), rabbit anti-NeuN antibody (1:200, ab104224, abcam), mouse anti-CD31 antibody (1:100, PA5-32321, Invitrogen), or mouse anti-MAP2 antibody (1:200, ab11267, abcam). On the following day, the slices were treated for an hour with Alexa Fluor 488 goat anti-mouse IgG or Alexa Fluor 488 goat anti-rabbit. After a final washing step with PBS, the sections were mounted onto glass slides. Images were captured by microscopy (FV3000, Olympus, Japan). Three-dimensional (3D) reconstruction of Iba-1 and GFAP positive cells within the peri-infarcted cortex was performed using Computer-based cell tracing software Neurolucida 360 (MBF Bioscience, Williston, VT, USA). NeuroExplorer (MBF Bioscience, Williston, VT, USA) was used to analyze 10 cells per mouse. Sholl analysis was used to determine branch tree morphology by placing 3D concentric circles in 5 mm increments starting at 5 mm from the soma.

### RNA pull-down assays

RNA pull-down assays were performed as previously described 54. In brief, 10^7^ cells were washed in ice-cold phosphate-buffered saline and lysed in 500 μl co-IP buffer (P0013F, Beyotime), and incubated for two hours at room temperature with 6 μg biotin-labeled circSCMH1 probe against endogenous or ectopically expressed circSCMH1. A total of 50 μl washed Streptavidin C1 magnetic beads (65002, Invitrogen) were added to each binding reaction and further incubated at room temperature for another hour. The beads were washed 5 times briefly with co-IP buffer. The bound proteins in the pull-down materials were analyzed by western blot. The mouse circSCMH1 probe sequence, which was biotinylated at the 5'-end, was 5'aaaTTGGAGGTGTGTAGGACTTTGGTGCCAGGTGG-3'.

### qPCR

qPCR was carried out using an Applied Biosystems qPCR system in accordance with our previous study. (StepOne, Thermo Fisher, USA) 18. Total RNA was extracted using TRIzol reagent (15596026, Invitrogen). CircRNAs and mRNAs were reverse transcribed using a HiScript Q RT SuperMix for qPCR Kit (R123-01, Vazyme) and quantified using SYBR Green qPCR Master Mix (Q141-02, Vazyme). β-actin was used as an internal control. Invitrogen synthesized the primers that were utilized to amplify circRNA and mRNA transcripts. The sequences of the primers are listed in Table S2 in the Supplement.

### Chromatin immunoprecipitation (ChIP) assay

The ChIP assay was conducted following the manufacturer's instructions (P2078, Beyotime). Briefly, the medium for cross-linking was immediately supplemented with new formaldehyde, and the final formaldehyde content was 1%. After 10 min of incubation at room temperature, the unreacted formaldehyde was quenched with 10 × glycine for 5 min at room temperature following incubation at room temperature for 10 min. Washed cells were scraped with cold PBS containing 1 mM PMSF and then centrifuged (1,000 × *g*, 2 min, 4 °C) to pellet the cells. The nuclei were extracted from the cell pellet using SDS lysis buffer containing 1 mM PMSF. DNA was sheared by sonication. The fragmented, cross-linked chromatin was diluted with Protein A + G Agarose/Salmon Sperm DNA and incubated for 30 min. Following centrifugation, the cross-linked chromatin was combined with antibodies targeting STAT5B (ab178941, Abcam), Histone H3 (9715S, Cell Signaling), and IgG (2729, Cell Signaling), and allowed to incubate overnight at 4 °C. The cross-linked protein/DNA complexes were reversed to release the bound DNA using elution buffer after being cleaned with a series of cold wash buffers (including a low-salt buffer, a high-salt buffer, an LiCl buffer, and finally, a TE buffer) and purified using DNA purification spin columns. Finally, the purified DNA was amplified via PCR to identify the promoter region containing the specific binding site. The sequences of the primers are listed in Table S3 in the Supplement.

### Cytoplasmic and nuclear protein extraction

Cytoplasmic and nuclear protein from HT-22 cells was prepared using the nuclear and cytoplasmic separation kit from Beyotime (P0028). Obtained supernatants were stored at -80 °C until use.

### Western blot analysis

Proteins were extracted in RIPA lysis buffer (P0013B, Beyotime), separated on sodium dodecyl sulfate polyacrylamide gels (10% and 12%), and electrophoretically transferred onto polyvinylidene fluoride membranes as previously mentioned 14. Rabbit anti-KMO antibody (1:1,000, 10698-1-AP, Proteintech), rabbit anti-OPA1 antibody (1:1,000, 27733-1-AP, Proteintech), rabbit anti-MFN2 antibody (1:1,000, 12186-1-AP, Proteintech), rabbit anti-DRP1 antibody (1:1,000, 12957-1-AP, Proteintech), rabbit anti-LC3B antibody (1:1,000, L7543, Sigma), rabbit anti-SQSTM1 antibody (1:1,000, 18420-1-AP, Proteintech), rabbit anti-COX4I1 antibody (1:1,000, 11242-1-AP, Proteintech), rabbit anti-TOM20 antibody (1:1,000, 11802-1-AP, Proteintech), rabbit anti-iNOS antibody (1:1,000, 18985-1-AP, Proteintech), mouse anti-GFAP antibody (1:1,000, 60190-1-Ig, Proteintech), mouse anti-PSD95 antibody (1:1,000, ab2723, Abcam), rabbit anti-Synaptophysin antibody (1:1,000, 17785-1-AP, Proteintech), rabbit anti-STAT5B antibody (1:1,000, ab178941, Abcam), rabbit anti-ZO-1 antibody (1:1,000, 21773-1-AP, Proteintech), rabbit anti-Occludin antibody (1:1,000, 13409-1-AP, Proteintech), rabbit anti-Histone antibody (1:1,000, 9715S, Cell signaling), or mouse anti-β-actin antibody (1:3,000, 66009-1-lg, Proteintech) were used to detect signals overnight at 4 °C. Membranes were then incubated with a HRP-conjugated affinipure goat anti-mouse or rabbit IgG(H + L) secondary antibody (1:2,000, SA00001-1 or SA00001-2, Proteintech) for an hour at room temperature. Signals were detected by chemiluminescence and imaged on a Tanon (Tanon 5200, Shanghai) digital image scanner. Quantification of individual protein bands was performed by densitometry using Image J software.

### Statistics

All data are presented as mean ± SEM. Comparisons between 2 groups were assessed with a 2-tailed Student's t-test. The homogeneity of variance was evaluated via Brown-Forsythe test. Comparisons among multiple groups (4 or more) were assessed with Two-way ANOVA followed by the Holm-Sidak post hoc test. Behavioral data collected at repeating time points (i.e., for a single animal at 5 time points) were analyzed using Two-way repeated measures ANOVA, followed by the Holm-Sidak post hoc test. The statistical analysis used for different experiments is indicated in the figure legends, and statistical significance was set at *P* < 0.05.

## Supplementary Material

Supplementary figures and tables 1-3.

Supplementary table 4: the results of metabolomics analyses.

## Abbreviations

3-HK: 3-hydroxykynurenine
ChIP: Chromatin Immunoprecipitation
COX4I1: cytochrome c oxidase subunit 4I1
circSCMH1: circular RNA SCMH1
circRNAs: circular RNAs
DAPI: 4',6-diamidino-2-phenylindole
DRP1: dynamin related protein 1
EVs: extracellular vesicles
GFAP: glial fibrillary acidic protein
GFP: green fluorescent protein
iNOS: inducible nitric oxide synthase
KMO: kynurenine 3-monooxygenase
KP: kynurenine pathway
Kyn: kynurenine
LC3B: microtubule associated protein 1 light chain 3 beta
LV: lentivirus
MAP2: microtubule associated protein 2
MFN2: mitofusin 2
OGD: oxygen glucose deprivation
OPA1: mitochondrial dynamin like GTPase OPA1
PSD95: postsynaptic density protein 95
PT: photothrombotic stroke
qPCR: quantitative polymerase chain reaction
RVG: rabies virus glycoprotein
SQSTM1: sequestosome 1
STAT5B: signal transducer and activator of transcription 5B
TOM20: translocase of outer membrane 20
Trp: tryptophan
ZO-1: tight junction protein 1
