# Supplementary material for: Circular RNA SCMH1 suppresses KMO expression to inhibit mitophagy and promote functional recovery following stroke
Source: Theranostics. 2024 Oct 28;14(19):7292–308. doi: 10.7150/thno.99323 (PMC11626939; doi:10.7150/thno.99323)
Supplement: Supplementary file 1 — Supplementary figures and tables 1-3. [file thnov14p7292s1.pdf]

Supplementary Materials for

Circular RNA SCMH1 suppresses KMO expression to inhibit mitophagy and functional recovery following stroke

Yu Wang[1], Ying Bai[1], Yang Cai[1], Yuan Zhang[1], Ling Shen[1], Wen Xi[1], Zhongqiu Zhou[1], Lian Xu[1], Xue Liu[1], Bing Han[1*], Honghong Yao[1,2,3*]

[1]Jiangsu Provincial Key Laboratory of Critical Care Medicine, Department of Pharmacology, School of Medicine, Southeast University, Nanjing, China;

[2]Co-innovation Center of Neuroregeneration, Nantong University, Nantong, China;

[3]Institute of Life Sciences, Key Laboratory of Developmental Genes and Human Disease, Southeast University, Nanjing, China

[*]Corresponding author:

Honghong Yao, Ph.D., Jiangsu Provincial Key Laboratory of Critical Care Medicine, Department of Pharmacology, School of Medicine, Southeast University, Nanjing, China; Tel: +8625 83272551; E-mail: yaohh@seu.edu.cn

Bing Han, Ph.D., Jiangsu Provincial Key Laboratory of Critical Care Medicine, Department of Pharmacology, School of Medicine, Southeast University, Nanjing, China; Tel: +8625 83272551; E-mail: hanb@seu.edu.cn

Figure S1

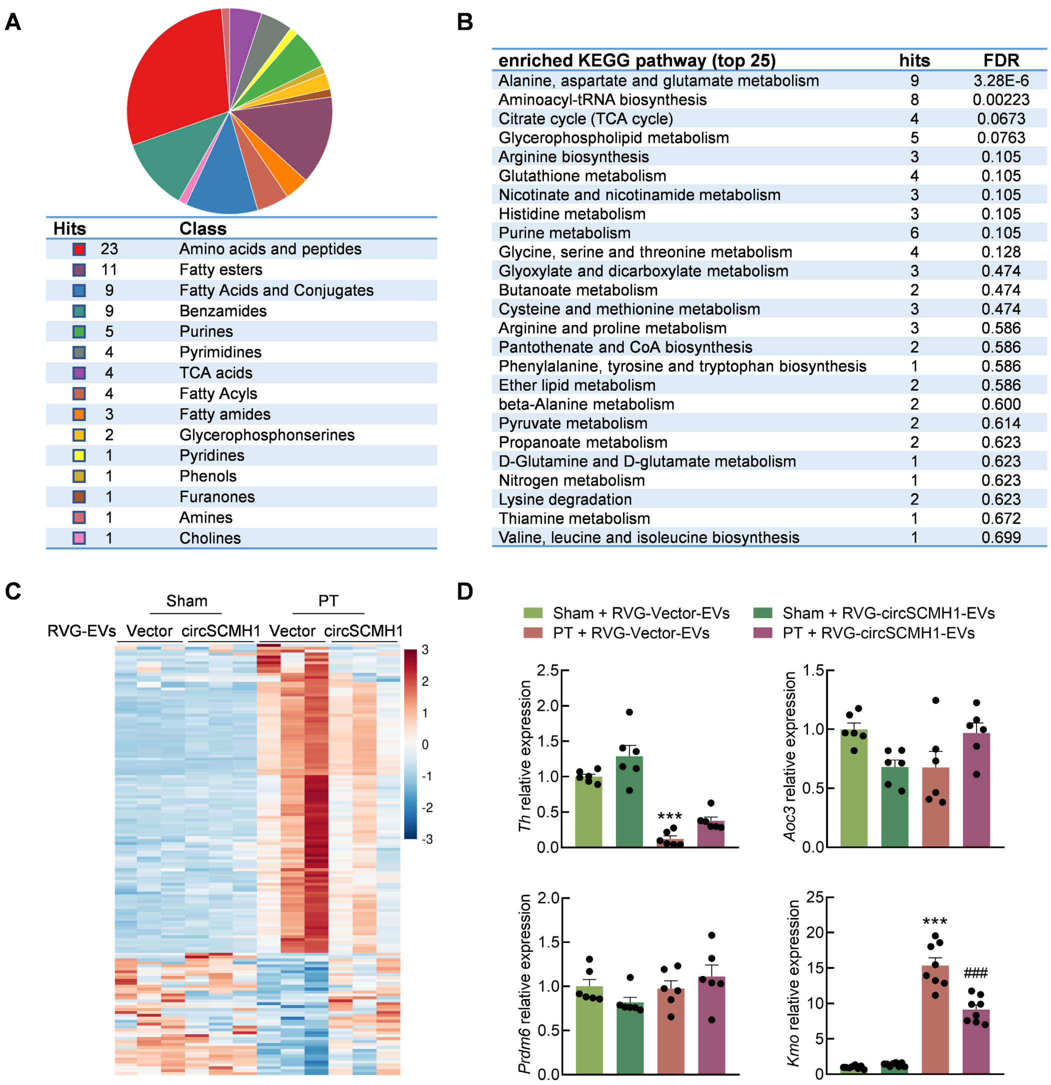

Figure S1. The selection of differential metabolites and differential genes. (**A**) Pie chart and table show the differential metabolites classification enrichment results. (**B**) Table show Metaboanalyst KEGG pathway enrichment of differential metabolites. (**C**) Heatmap analysis of 148 differential genes in PT and sham mice with overexpressed circSCMH1 and Vector (n = 3). (**D**) Relative expression of 4 genes involved in amino acid metabolism in peri-infarcted cortex tissue of PT mice. n = 6 samples/group. ****P* < 0.001 versus Sham + RVG-Vector-EVs group; ###*P* < 0.001 versus PT + RVG-Vector-EVs group using 2-way ANOVA followed by Holm-Sidak post hoc multiple

comparisons test. EVs, extracellular vesicles; KEGG, Kyoto Encyclopedia of Genes and Genomes; PT, photothrombotic stroke; RVG, rabies virus glycoprotein.

Figure S2

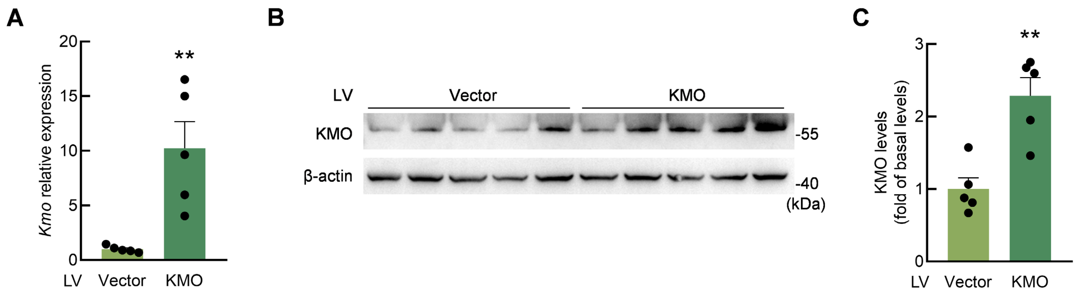

Figure S2. Expression of KMO in cortex two weeks later after KMO-GFP lentivirus microinjection. (**A**) qPCR analysis of the expression of *Kmo* in mice cortex after LV-KMO microinjection. All data were presented as mean ± SEM of five independent experiments. $^{**}P < 0.01$ versus LV-Vector group using Student's t-test. (**B, C**) Western blot analysis of KMO expression after LV-KMO treatment in mice cortex. $^{**}P < 0.01$ versus the LV-Vector group using Student's t-test. GFP, green fluorescent protein; KMO, kynurenine 3-monooxygenase; LV, lentivirus; qPCR, quantitative polymerase chain reaction.

Figure S3

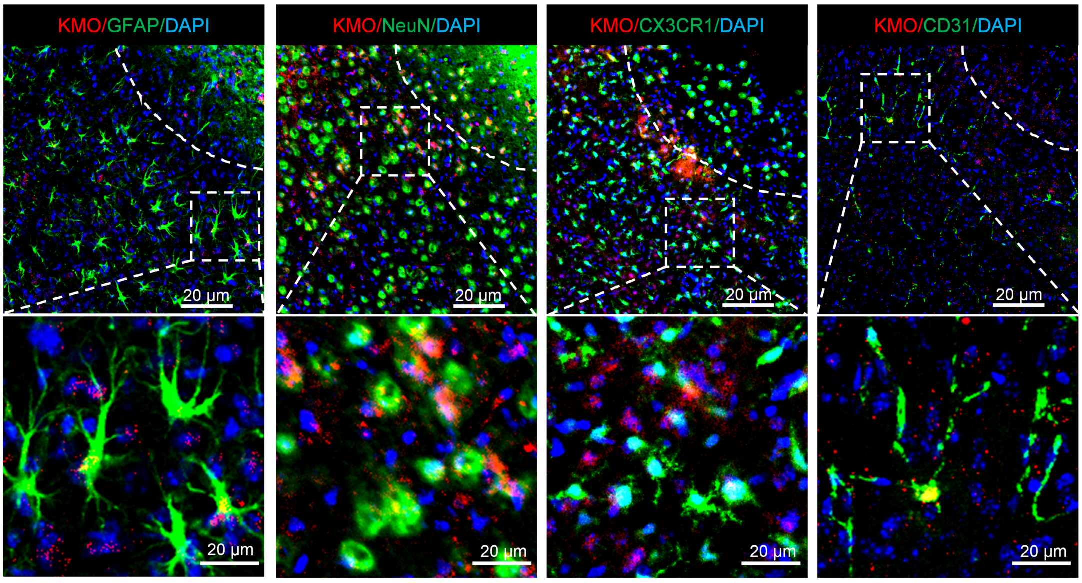

Figure S3. The distribution of KMO in mouse brain. The images of the distribution of KMO in neurons (NeuN^{+}), astrocytes (GFAP^{+}), microglia (CX3CR1^{GFP} mice) and endothelial cell (CD31^{+}) on day 3 after PT treatment. KMO, kynurenine 3-monooxygenase; DAPI, 4′,6-diamidino-2-phenylindole; GFAP, glial fibrillary acidic protein.

Figure S4

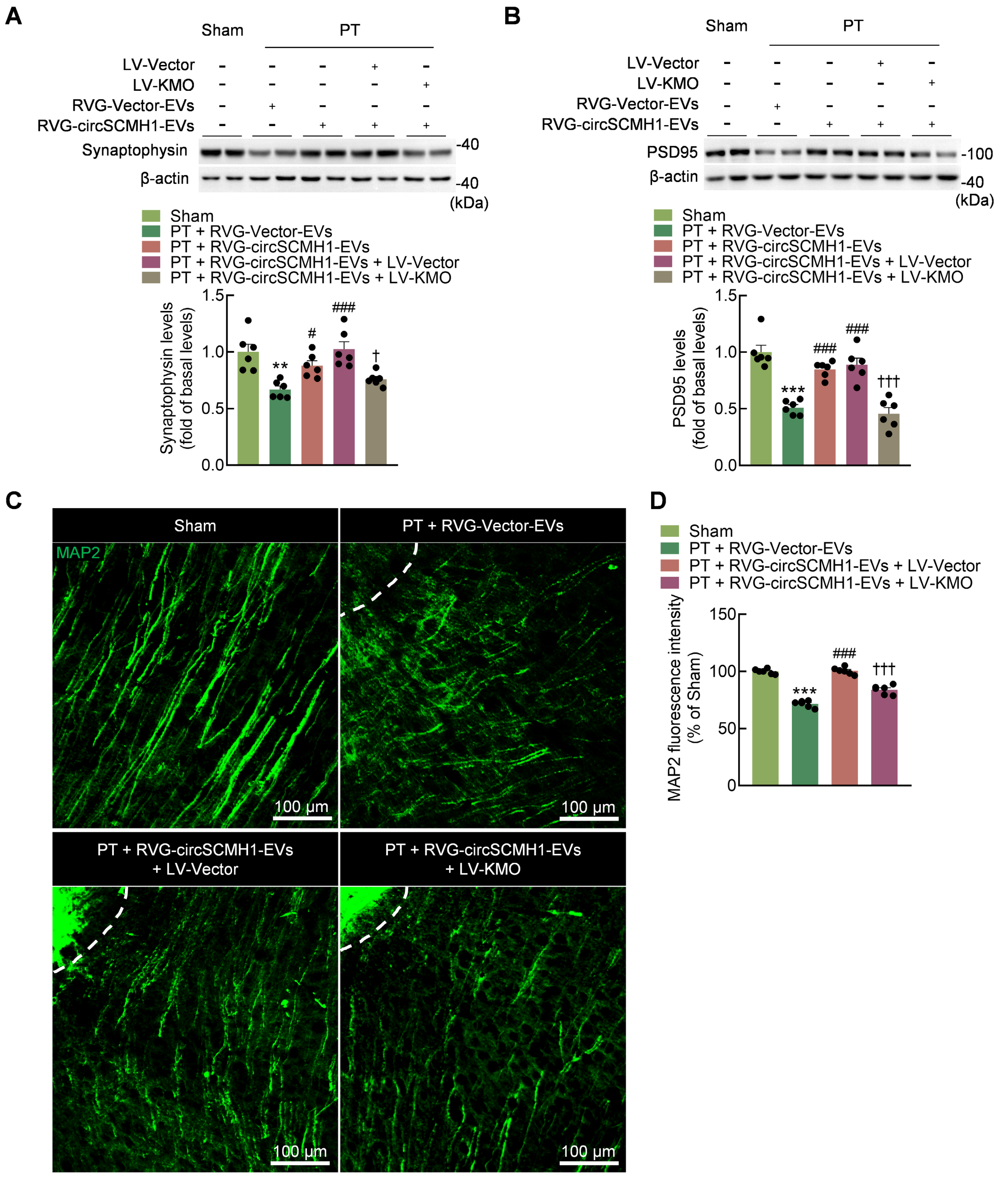

Figure S4. Neuroplasticity remodeling is enhanced by the inhibition of KMO expression. (**A, B**) Western blotting analysis of Synaptophysin and PSD95 expression after LV-KMO and RVG-circSCMH1-EVs treatment in PT mice. Two representative immunoblots were presented from 6 mice/group. **P < 0.01, ***P < 0.001 versus the Sham group; #P < 0.05, ###P < 0.001 versus the PT + RVG-Vector-EVs group; †P < 0.05, †††P < 0.001 versus the PT + RVG-circSCMH1-EVs + LV-Vector group using

two-way ANOVA followed by the Holm-Sidak post hoc multiple comparison test. (**C, D**) Representative images of MAP2 staining and the analysis. Representative images of MAP2 staining on day 28 after PT in mouse brain (C) and the analysis of MAP2 fluorescence intensity (D). n = 6 mice/group. ****P* < 0.001 versus the Sham group; ###*P* < 0.001 versus the PT + RVG-Vector-EVs group; †††*P* < 0.001 versus the PT + RVG-circSCMH1-EVs + LV-Vector group using two-way ANOVA followed by the Holm-Sidak post hoc multiple comparison test. EVs, extracellular vesicles; KMO, kynurenine 3-monooxygenase; LV, lentivirus; MAP2, microtubule associated protein 2; PSD95, postsynaptic density protein 95; PT, photothrombotic stroke; RVG, rabies virus glycoprotein.

Figure S5

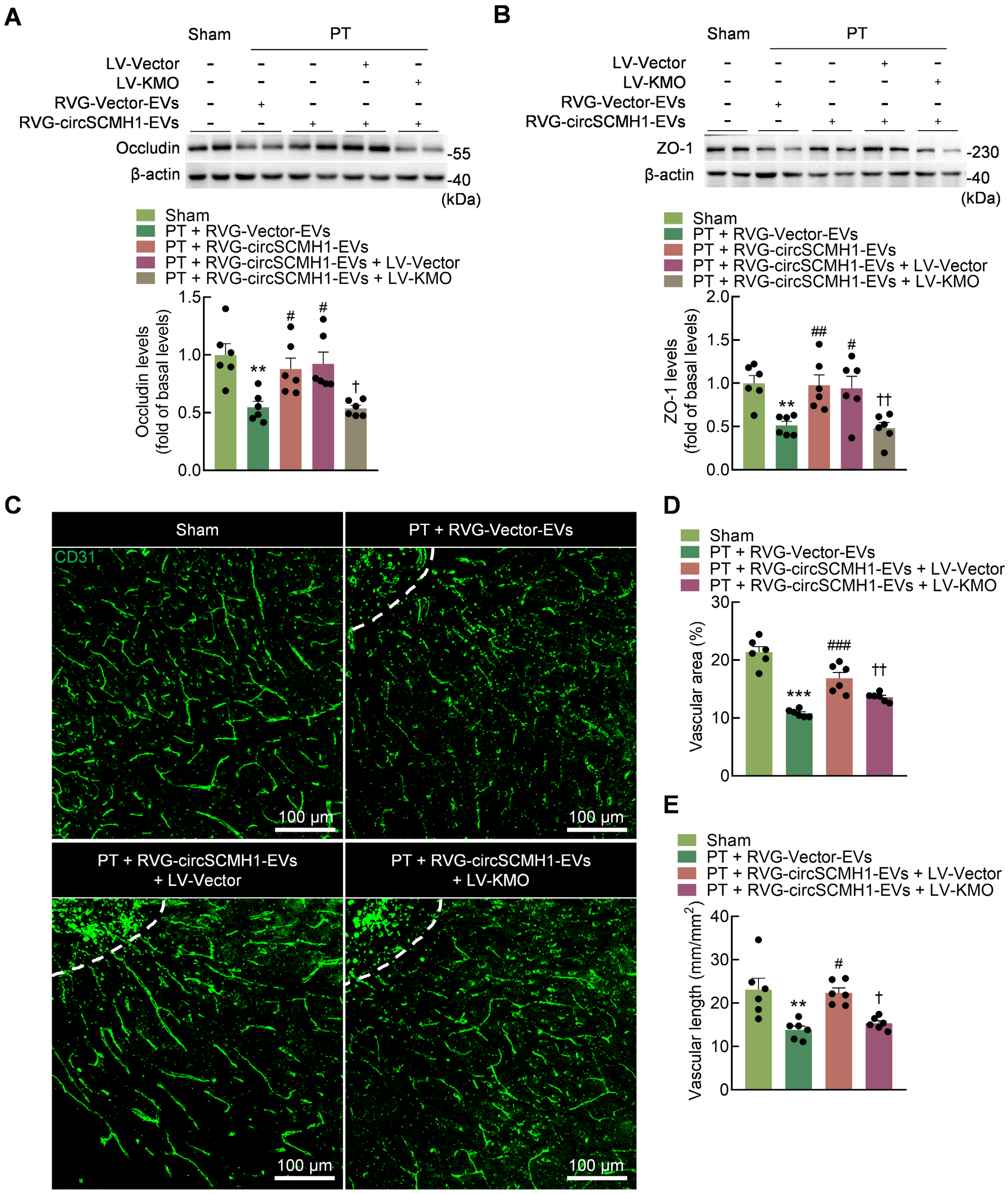

Figure S5. Vascular remodeling is enhanced by the inhibition of KMO expression. (**A, B**) Western blotting analysis of Occludin and ZO-1 expression after LV-KMO and RVG-circSCMH1-EVs treatment in PT mice. Three representative immunoblots were presented from 6 mice/group. ***P* < 0.01 versus the Sham group; #*P* < 0.05, ##*P* < 0.01 versus the PT + RVG-Vector-EVs group; †*P* < 0.05, ††*P* < 0.01 versus the PT + RVG-circSCMH1-EVs + LV-Vector group using two-way ANOVA followed by the Holm-

Sidak post hoc multiple comparison test. (**C-E**) Representative images with CD31 staining showing blood vessels in the peri-infarct cortex on day 28 after PT in mice, followed by the analysis of vascular area fraction and vascular length. n = 6 mice/group. ***P* < 0.01, ****P* < 0.001 versus the Sham group; #*P* < 0.05, ###*P* < 0.001 versus the PT + RVG-Vector-EVs group; †*P* < 0.05, ††*P* < 0.01 versus the PT + RVG-circSCMH1-EVs + LV-Vector group using two-way ANOVA followed by the Holm-Sidak post hoc multiple comparison test. EVs, extracellular vesicles; KMO, kynurenine 3-monooxygenase; LV, lentivirus; PT, photothrombotic stroke; RVG, rabies virus glycoprotein; ZO-1, tight junction protein 1.

Table S1. Binding score of transcription factors with circSCMH1.

Protein	RNA	Interaction Propensity	Z-score	RBP propensity	RNA-Binding Domains	Strand
**STAT5B**	circSCMH1	44.91	1.91	0.28	4	+
**MafG**	circSCMH1	15.91	0.15	0.59	1	+
**VDR**	circSCMH1	10.09	-0.21	0.36	2	+
**Zic2**	circSCMH1	9.77	-0.23	1	3	+
**HOXD9**	circSCMH1	6.35	-0.44	0.88	2	+
**Sox2**	circSCMH1	6.19	-0.44	0.67	3	+
**Zic3**	circSCMH1	2.6	-0.66	1	3	+

Table S2. The primer sequences used for RT-PCR analysis.

List of oligonucleotide sequences	5'-3'
circSCMH1 (mouse)-F	CTACTGGTGCCGCTTTGACT
circSCMH1 (mouse)-R	GGCACCTGTCAATCCAACGA
Actb (mouse)-F	GGCTGTATTCCCCTCCATCG
Actb (mouse)-R	CCAGTTGGTAACAATGCCATGT
Kmo (mouse)-F	CAAGGAATGAATGCGGGCTT
Kmo (mouse)-R	ACCTAGAGTTGACATGCGCT
Th (mouse)-F	GTGGAGACAGAACTCGGGAC
Th (mouse)-R	AATGAACCTTGGGGACGTGA
Prdm6 (mouse)-F	CGACTGGCATCTCTGGAAGT
Prdm6 (mouse)-R	ACACTGGTAGGGTCTGTCGG
Aoc3 (mouse)-F	AACAAGTGGGGTCATAGGCG
Aoc3 (mouse)-R	CAGCCAAGTGATACCTCCCC

Table S3. The primer sequences used for CHIP-PCR analysis.

List of oligonucleotide sequences	5'-3'
STAT5B (mouse)-F	CTGGTCAGTCTAGTTACACCTCT
STAT5B (mouse)-R	TGTGGGCTTGTTGCCTTAAC
